# A Novel Approach for Brain Tumor Classification Using an Ensemble of Deep and Hand-Crafted Features

**DOI:** 10.3390/s23104693

**Published:** 2023-05-12

**Authors:** Hareem Kibriya, Rashid Amin, Jinsul Kim, Marriam Nawaz, Rahma Gantassi

**Affiliations:** 1 Department of Computer Sciences, University of Engineering and Technology, Taxila 47050, Pakistan; 2 Department of Computer Sciences, University of Chakwal, Chakwal 48800, Pakistan; 3 School of Electronics and Computer Engineering, Chonnam National University, 300 Yongbong-dong, Buk-gu, Gwangju 500757, Republic of Korea; 4 Department of Software Engineering, University of Engineering and Technology, Taxila 47050, Pakistan; 5 Department of Electrical Engineering, Chonnam National University, Gwangju 61186, Republic of Korea

**Keywords:** brain tumor, artificial intelligence, GLCM, KNN, VGG16

## Abstract

One of the most severe types of cancer caused by the uncontrollable proliferation of brain cells inside the skull is brain tumors. Hence, a fast and accurate tumor detection method is critical for the patient’s health. Many automated artificial intelligence (AI) methods have recently been developed to diagnose tumors. These approaches, however, result in poor performance; hence, there is a need for an efficient technique to perform precise diagnoses. This paper suggests a novel approach for brain tumor detection via an ensemble of deep and hand-crafted feature vectors (FV). The novel FV is an ensemble of hand-crafted features based on the GLCM (gray level co-occurrence matrix) and in-depth features based on VGG16. The novel FV contains robust features compared to independent vectors, which improve the suggested method’s discriminating capabilities. The proposed FV is then classified using SVM or support vector machines and the k-nearest neighbor classifier (KNN). The framework achieved the highest accuracy of 99% on the ensemble FV. The results indicate the reliability and efficacy of the proposed methodology; hence, radiologists can use it to detect brain tumors through MRI (magnetic resonance imaging). The results show the robustness of the proposed method and can be deployed in the real environment to detect brain tumors from MRI images accurately. In addition, the performance of our model was validated via cross-tabulated data.

## 1. Introduction

The brain is an important organ that oversees entire bodily functions and is a vital organ that is a central part of the nervous system. It can be affected by one of the most lethal brain diseases, called a brain tumor, caused by an unusual growth of cell proliferation within the brain. These tumors cause brain damage and dysfunctions, which can be very lethal if left untreated for a long time. The World Health Organization (WHO) has predicted an annual 5% increase in brain tumors [1]. Another report declares brain tumors the 10th leading cause of mortality among humans. According to estimates, at least 18,600 individuals will die from a deadly brain or central nervous system (CNS) tumor this year [2]. Thus, on-time and accurate tumor diagnosis can increase the patient’s chances of survival.

The patient’s health depends on a prompt and precise diagnosis of a brain tumor because the stage and kind of tumor influence the course of treatment. As tumors differ in size, location, and shape, identifying one can be challenging. An inaccurate or delayed diagnosis may lower the likelihood of a patient surviving a brain tumor. Medical professionals have historically used visual evaluation of medical imaging and precise tumor location tracing to diagnose brain tumors. These medical images are from MRIs, computed tomography (CT), and positron emission tomography (PET). Radiologists and medical experts frequently utilize MRI scans to identify brain tumors because they produce high-quality images of soft tissues [3]. Because of the surrounding healthy tissues, the tumor margins are frequently hazy when manually detecting tumors through optical inspection. Because of this, manually identifying tumors takes a long time and usually results in incorrect tumor diagnosis. Images that are noisy for various reasons, such as medical image acquisition techniques or variations in imaging equipment, are another cause of tumor misidentification [4]. Usually, biopsies are carried out to ascertain whether the tissue is malignant or benign. Determining the cancer is a painful and time-consuming technique. Consequently, automated technologies are replacing traditional approaches due to the complexity of current approaches [5,6,7].

Recognizing and categorizing brain tumors early for a patient to receive the proper treatment is crucial. Thanks to technological advancements, professionals may treat patients properly using automated healthcare systems. More research is being presented to address the challenges in medical image identification as machine learning and artificial intelligence technologies improve [8]. These automated methods effectively diagnose brain tumors and aid medical personnel in deciding appropriate treatment procedures [9]. Given that the human eye cannot distinguish between the numerous shades of grey in MRI images, these systems are extremely useful for detecting even the slightest color changes. A persistent problem in medical image analysis is storing and evaluating large amounts of medical data. However, the existing systems cannot effectively manage the noticeable increase in data volume in the medical sector. Currently, contemporary machine learning algorithms frequently utilize big data approaches to analyze medical image data. The development of new technologies, especially machine learning and artificial intelligence, has substantially impacted the medical industry because it has provided medical departments with an essential tool to get second opinions with much higher precision. The robust automated frameworks work best where radiologists want to minimize the possibility of biopsy or inspect tumor depth or type [10].

Although numerous works have been proposed for the effective recognition of brain tumors, there is room for performance improvement. Employing only deep learning algorithms demands vast quantities of labeled data, while the supervised approach to classifying a brain tumor has much potential; however, it takes specialized knowledge to extract the best characteristics and selection methods. The paper proposes a novel and fully automated brain tumor identification and classification method that combines hand-engineered and deep features to fill this gap. The novel FV is an ensemble of hand-crafted features based on the GLCM (gray level co-occurrence matrix) and deep features based on VGG16. These FVs, when combined, increase the discriminating ability of the proposed system, allowing for accurate tumor detection even in the presence of backgrounds, ill-defined tumor boundaries, skulls, and other MRI artifacts. The proposed FV is then classified using SVM or support vector machines and the k-nearest neighbor classifier (KNN). The suggested framework performs better than the existing methods at quickly and accurately detecting brain cancers. The following are the primary contributions of the suggested system:

- We present a novel method based on an ensemble of deep and hand-crafted features to classify brain tumors in MR images.
- Per our knowledge, this is the first-ever study based on a feature-level ensemble of VGG16 and GLCM features to classify brain tumors.
- Our framework consists of three main core steps: deep feature extraction via CNN, that is, through the VGG16 model, hand-crafted feature computation via GLCM, creating an ensemble vector of these FVs, and finally, classification using SVM and KNN.
- The proposed method effectively classifies brain tumors because the fusion of the GLCM and deep FV computes an effective set of image features, resulting in better discrimination of tumor and normal images.
- The results indicate the efficacy of the presented approach as compared to existing methodologies.

## 2. Related Work

Early tumor detection and classification of brain tumors are required for efficient patient therapy. Thanks to impressive technological advancements, specialists may treat patients more effectively using automated healthcare systems. Researchers have recently developed several methods for classifying brain tumors that use ML and DL-based algorithms. New artificial intelligence and deep learning technologies have substantially impacted medical picture processing, notably in an illness diagnosis. This section examines the research on methods for classifying brain tumors using ML and DL-based algorithms. Anaraki et al. [7] used Genetic Algorithms to perform brain tumor categorization in the pituitary, glioma, and meningioma malignancies from MRI images. The system attained 94.2% accuracy. However, the algorithm could not identify an ideal CNN design, leading to poor performance. Using the complete volumetric T1-Gado MRI sequence from the Brats 2018 dataset, Mzoughi et al. [11] presented DL architectures to grade glioma tumors based on severity. The authors, in their framework, incorporated local and global features with lower weights by using small kernels that resulted in an accuracy score of 96%. Sejuti et al. [12] presented a CNN-SVM-based method to identify and classify brain tumors in MRI images and attained 97.1% accuracy. Whereas the authors of Abiwinanda et al. [13] designed five different CNN frameworks to detect tumors in the brain. The system obtained 84.1% accuracy. However, it is worth mentioning that relatively basic CNNs cannot extract complex high-level features, leading to mediocre overall accuracy. Due to this, the CNNs in [11,12,13] have resulted in poor performance because of very simple architectural designs.

To identify an ideal CNN design with lower computation costs for the classification of brain tumors, a hierarchical deep learning-based brain tumor classification technique was presented by Khan et al. [14]. The study obtained 92% accuracy on the Kaggle database. However, the system must be evaluated rigorously to view its efficacy in real-case scenarios. Alanazi et al. [15] demonstrated a brain tumor detection system composed of 22 layers, resulting in 96.8% accuracy. However, only a few imaging samples are used to conduct the study; hence, a thorough evaluation is crucial. Afshar et al. [16] used capsule networks that achieved an accuracy of 90.8%. However, one of the limitations of capsule networks is their sensitivity to image backgrounds. These architectures tend to perform much better in the case of segmentation image input. Noureen et al. [17] fine-tuned the Inception-v3 and Xception models for deep CNN features extraction, whereas an ensemble of different classifiers, i.e., SVM, KNN, and random forest (RF), was used to classify the images. The system obtained 93.3% overall accuracy. In another study, the authors of Swati et al. [6] trained and evaluated transfer-learned AlexNet architecture to identify brain tumors from MR images, resulting in 89.9% overall accuracy. However, due to low overall accuracy, the systems in [6,17] must be evaluated before deploying in real-world scenarios.

Kang et al. [18] presented a feature ensemble method using vectors obtained from DenseNet169, Inception-v3, and ResNeXt50. The system resulted in 98.5% accuracy. In another study, the authors of Waghmere et al. [19] developed a brain tumor classification method using VGG16 architecture. The system resulted in 95.7% accuracy on preprocessed and augmented MRI images. However, the studies mentioned in [18,19] are computationally complex. Some of the authors also proposed segmentation before classification methods. For instance, . Naser et al. [20] employed a U-Net architecture to segment the tumors and VGG16 to perform classification. The study was performed on the TCIA dataset and achieved almost 92% accuracy. Masood et al. [21] employed Mask RCNN to classify brain tumors. In the first stage, they localized the tumor region using bounding boxes, and in the next stage, they classified the tumor. A multimodal tumor classification system built on CNN was developed by Sajjad et al. [22]. They used input cascade CNN to first segment the MRIs, then classified them with 94.5% accuracy using a tuned VGG-19. However, the systems are computationally expensive as they perform segmentation before the classification phase.

On the other hand, various researchers have proposed ML-based algorithms to detect brain tumors using hand-crafted feature extraction and classification. Amin et al. [23] extracted and classified hand-crafted features using GLCM and SVM, respectively, and obtained 97% accuracy. Kaplan et al. [24] used Local Binary Pattern (LBP) for feature extraction KNN for classification. They achieved 95.5% overall accuracy. Bahadure et al. [25] segmented the tumor region and extracted hand-crafted features using GLCM. The system obtained 96.5% accuracy via SVM. Garg et al. [26] applied the Otsu threshold to MRI images and extracted GLCM features. They performed classification via SVM and KNN with 97.3% accuracy. Minz et al. [27] extracted GLCM features from segmented images and performed classification via AdaBoost. The system obtained the highest accuracy of 89.9%. However, these systems require manual segmentation of tumor regions before the feature extraction and classification phase, thus increasing the system’s complexity. Raja et al. [28] used information-theoretic measures and Bayesian fuzzy clustering techniques to segment images, whereas the nonlocal mean filter for image de-noising and scattering transform. They used Tsallis entropy as feature extractors. Finally, a hybrid DAE approach was deployed for the classification of tumors. However, the computation required for this method is time-consuming and inefficient. Ali et al. [29] proposed an approach by employing two deep learning approaches named the GoogleNet and YOLO models to recognize the brain tumors from the MRI samples. They attained the highest accuracy of 97% with the first model. Another approach was proposed in [30] that linked RoB and different AI-based architectural clusters in various DL frameworks to investigate them and attain improved analysis results.

In brain tumor segmentation, the k-nearest neighbor algorithm is an object classification technique using the closest learning instances in the problem space. However, KNN is one type of instance-based learning, or lazy learning, in which the function is only locally approximated, and all computations are deferred until classification. The k-nearest neighbor algorithm is one of the simplest machine learning algorithms: an object is classified by a majority vote of its neighbors, with the object assigned to the most frequent class among its k-nearest neighbors. The k-NN algorithm functions as follows:

- Compute the Euclidean or Mahalanobis distance between the target and sampled plots.
- Arrange samples according to the calculated distances.
- Select the optimal k-nearest neighbors heuristically based on the RMSE obtained by the cross-validation technique.
- Compute a weighted average of the inverse distance to the k multivariate nearest neighbors.

The support vector machine was selected because it is an interesting framework from a machine-learning perspective. Specifically, SVM is a linear or non-linear classifier, a mathematical function capable of distinguishing two different types of objects. Such objects are divided into classes, which should not be confused with an implementation [23,24,25,26,27]. Ref. [31] proposed the classification of a brain tumor in brain MRI images using an image mining technique. The median filtering and features preprocessed the MRI images have been extracted using the texture feature extraction technique. Decision tree classification and the interclass relationship in text classification are used to improve the efficiency of traditional mining methods. The system used an SVM classifier, which gives 83% accuracy. Classification of brain tissues through MRI using a hybrid approach of GA and SVM is proposed by Ahmed Kharrat et al. [32]. The features are extracted by the spatial gray level dependence method called SGLDM. The proposed system gives a good accuracy of about 85.22% [33], and proposed morphological operations to the image, then extracted the features. Analysis result from MLPNN and SVM shows these operations can improve classification results in symmetry and grayscale features but reduce results in texture features. Using SVM, the system gets a better result than MLPNN and RBFNN. Because of the brain’s symmetrical structure, symmetrical features have better accuracy, and texture features have lower accuracy.

## 3. Proposed Methodology

**Figure 1:**
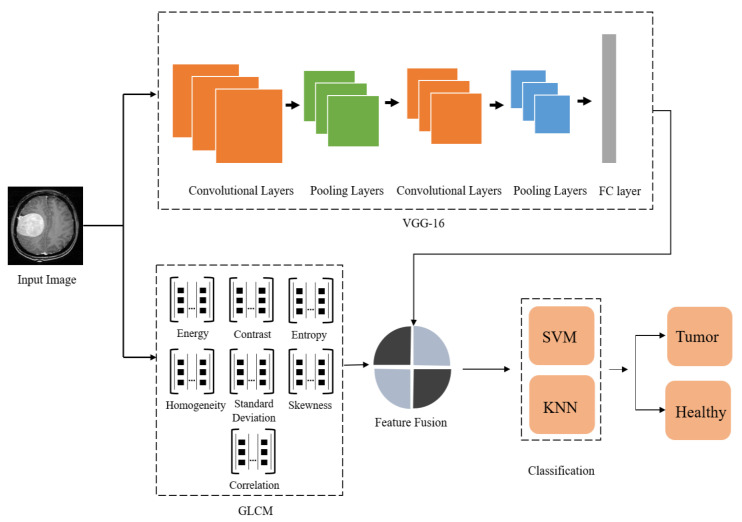
Block diagram of the proposed method indicating all the steps in detail.

This section discusses the proposed brain tumor classification framework in detail. The proposed methodology consists of 4 main steps (as shown in Figure 1): image preprocessing, deep and hand-crafted feature extraction, feature level ensemble of extracted FVs, and finally, tumor classification. Initially, the dataset images are scaled to 225 × 225 to match CNN’s input layer size. We extracted hand-engineered and deep features in the feature extraction phase using GLCM and VGG-16, respectively. We presented a feature ensemble methodology that combines both FVs (hand-crafted + deep), which is then classified via SVM and KNN. Feature ensemble combines features from multiple architectures in a single FV, thus eliminating the requirement to use a single FV obtained from low performing model. Feature ensemble leads to better classification outcomes as the new FV is more elaborate and informative than a single one. This method can, thus, aid in developing an efficient model for precisely detecting and classifying brain tumors, which radiologists can use to get a second opinion.

### 3.1. Feature Extraction

Feature extraction collects shape, texture, and color-related information from an image vital for representation. Obtaining optimal features from MRI is a very challenging task [34]. Feature extraction converts raw data into numerical representations while retaining the original information, which is then processed. These features can be extracted using automated techniques (i.e., DL) or manual methods (i.e., hand-engineered feature extraction). However, manual feature extraction requires a detailed understanding of extracting only those significant features for a certain issue. A firm understanding of the context or domain can frequently help decide which attributes might be beneficial. Scientists and researchers have spent years researching strategies for extracting and selecting optimal features from images, signals, audio/video, or text.

On the contrary, DL frameworks automate the feature extraction process without human intervention [35]. The proposed framework extracts hand-crafted features via GLCM and deep CNN features from the fully connected (FC7) layer of VGG16 architecture. We extracted both hand-engineered and deep features, which are later fused to form a robust, more discriminative feature vector than the independent vector. The details of extracted feature vectors are provided below:

#### 3.1.1. Deep Feature Extraction Using VGG-16

Deep learning is a subset of ML that uses multiple layers of neurons with complex architecture or non-linear processes to simulate high-level data abstractions. With the expansion in data volume and computational capacity, neural networks with more complicated topologies have received attention from academia to industry. Application-wise, deep learning has made significant strides in voice and image categorization, advancing artificial intelligence and human-computer interaction [36].

One of the widely used DL frameworks in a convolutional neural network (CNN) is a feed-forward ANN that employs convolutions in at least one of its layers. It drew inspiration from biological brain networks. CNNs combine ANN with discrete convolutions to extract robust features from the database. These architectures are very effective in identifying and classifying 2D data, i.e., images and videos. These networks take input directly from data (usually images/videos) and automate feature extraction/classification phases, thus saving time compared to conventional image recognition methods. Moreover, exploring robust deep features is crucial in precisely and accurately identifying images (including brain tumors). Due to their benefits, CNNs are known to achieve a state-of-the-art performance when used as deep feature extractors because of the capability to notice minute changes in images and capturing in the form of features [37,38].

**Figure 2:**
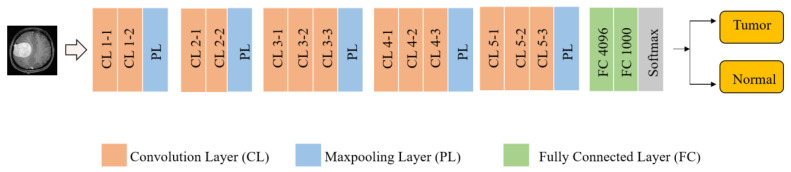
Architectural details of the VGG16 model.

In this paper, we used VGG-Net as our deep feature extractor. The framework was presented in 2014 by Karen Simonyan and Andrew Zisserman. The architecture comprised 138 million parameters and was ranked second in ILSVRC in 2014 [39]. The framework is widely employed in image classification due to its robust feature extraction and lightweight architecture [39]. The architecture of VGG-16 is illustrated in Figure 2. The first layer of the framework holds an input image of size 224 × 224. Next to the input layer is a convolution layer (CL) composed of different convolution filters responsible for convolving input images with kernels to obtain feature maps. Multiple stacked-up CLs improve the ability to learn hidden features. CLs are usually followed by an activation function (usually ReLU) that is applied to saturate the generated output. The next layer in this architecture is the pooling layer (PL) which aims to reduce the size of the feature maps to decrease the computational load to the next stages. The FC layer is responsible for producing the class scores from the activations for classification. The VGG-16 architecture comprises 13 CLs with multiple filters of size 3 × 3 throughout the entire network and 5 max-pooling layers. The last three FC layers result in 4096, 4096, and 1000 features [40,41,42]. The motivation behind using VGG-16 is that the CLs have a kernel size of 3 × 3 with a stride of 1 throughout the network, unlike other CNN models (such as ZF-Net, AlexNet) with a kernel size of 7 × 7 and 11 × 11 with 4 to 5 strides in the initial layers. Usually, the larger strides ignore important patterns in the MR images, whereas a larger kernel size increases the number of parameters.

#### 3.1.2. Hand-Crafted Feature Extraction Using GLCM

We also extracted hand-engineered features using GLCM. The main difference between deep features and hand-engineered feature vectors is that hand-crafted features are created in advance by humans and extract a specific set of desired features. In contrast, architecture learns CNN-based features from the provided data [43]. It may be noted that we extracted texture-based hand-crafted features (GLCM) that examine the spatial correlation of pixels among each other. These features are robust in determining a slight change in pixel intensities and can precisely differentiate between tumorous tissues and healthy tissues.

Harlick or GLCM [44] are texture-based features that extract second-order statistical texture features from gray-level images. These features focus on a specific position of the pixels relative to the other pixel, as the image texture usually contains important information regarding the structural arrangement of surfaces. GLCM is proven to produce robust results in different fields, including medical image diagnosis [45], as they can help diagnose brain tumors from MRIs by detecting minor changes in pixel variations. We extracted 7 textural features from MRI images using GLCM: energy, contrast, entropy, homogeneity, shade, and prominence. The hand-crafted features are discussed in this section. We extracted energy which measures the number of pixel repetitions in an image. When the image is homogenous, energy values will be higher, indicating textural uniformity. Energy is computed as in Equation (1). Here *m* and *n* denote the size of the GLCM matrix, whereas pixels are represented by *x*, *y*

(1) $$Energy=\sqrt{\overset{m-1}{\underset{x=0}{\sum }}\overset{n-1}{\underset{y=0}{\sum }}\;f^{2}\left(x,y\right)}$$

Another feature extracted from the GLCM matrix is contrast, which measures the intensity variation of a pixel and its neighboring pixels in an image. The contrast value will be higher when there is a large variation in image pixels. It can be defined as in Equation (2). Here GLCM matrix is denoted by *m* and *n*, whereas image pixels are represented by *x* and *y*.

(2) $$Contrast=\overset{m-1}{\underset{x=0}{\sum }}\overset{n-1}{\underset{y=0}{\sum }}{\left(x-y\right)}^{2}\;f\left(x,y\right)$$

We also calculated entropy which measures the randomness of pixel values in a textural image. Homogenous images possess a higher entropy value than inhomogeneous images, which have a lower entropy value. Entropy can be calculated as in Equation (3). The pixel intensities are represented by *x* and *y*, whereas m and n indicate the GLCM matrix.

(3) $$Entropy=-\overset{m-1}{\underset{x=0}{\sum }}\overset{n-1}{\underset{y=0}{\sum }}f\left(x,y\right)log_{2}f\left(x,y\right)$$

Inverse difference moment (IDM) determines the similarity and closeness between the pixels of an image to check if the image is textured or non-textured. The IDM’s value will be higher in the homogenous images than in non-homogenous images. It can be calculated as in Equation (4)

(4) $$IDM=\overset{m-1}{\underset{x=0}{\sum }}\overset{n-1}{\underset{y=0}{\sum }}\frac{1}{1+{\left(x-y\right)}^{2}}\;f\left(x,y\right)$$

Standard deviation (SD) describes probability distribution in an observed population. A higher SD value signifies better contrast and intensity in an image. It can be defined as in Equation (5). Here *x* and *y* denote pixel values of MRI images, whereas *m* and *n* determine the GLCM matrix size.

(5) $$SD=\sqrt{\left(\frac{1}{mxn}\right)\overset{m-1}{\underset{x=0}{\sum }}\overset{n-1}{\underset{y=0}{\sum }}\left(f\left(x,y\right)-M^{2}\right)}$$

Skewness measures symmetry between image pixels. It will possess lower values when an image is less asymmetric. It is calculated in Equation (6). Pixel intensities are depicted using *x* and *y*. In contrast, the GLCM matrix is denoted by *m*, *n*.

(6) $$Skewness=\left(\frac{1}{mxn}\right)\frac{\sum^{\;}(f\left(x,y\right)-M^{3}}{\partial^{3}}$$

Correlation defines the spatial dependencies or relations between a pixel and its neighborhood. It can be calculated as in Equation (7), where *x* and *y* denote pixel intensities, whereas *m* and *n* show the size of the GLCM matrix.

(7) $$Correlation=\frac{\sum_{x=0}^{m-1}\sum_{y=0}^{n-1}\left(x,y\right)f\left(x,y\right)-M_{x}M_{y}}{\partial_{x}\partial_{y}}$$

### 3.2. Feature Ensemble

Because the performance of an ML classifier is strongly dependent on the input FV, establishing an approach to create a discriminative FV from MRIs is critical for effective tumor classification. Ensemble learning improves the system’s performance by merging several FVs in a single predictive FV. It also helps avoid the risk of utilizing an individual FV obtained from one model with poor performance. Depending on the integration, ensemble learning falls into two specific categories, i.e., feature-level ensemble and classifier-level ensemble. At the classifier level, ensemble integration is performed on output sets obtained from classifiers using voting methods to determine the final result. On the contrary, in a feature-level ensemble, the FVs are integrated and fed to the classifiers for the final result. This type of integration yields far better results since the FVs obtained from varying architectures are concatenated, which is much more informative than individual ones because of various features that determine boundaries, edges, shapes, and changes in intensities. Hence, we used this study’s feature-level ensemble method to combine deep and hand-engineered FVs. Independent FV acquired via GLCM is defined in Equation (8) which shows the 7 textural features extracted from MRI images, whereas Equation (9) shows 4096 deep features obtained from MRI images using VGG16. These features are then integrated into a novel FV mathematically given in Equation (10). Equation (10) shows the concatenation of both feature vectors computed with the GLCM, and VGG16 models, which results in a novel FV called ensemble vector (EV) comprised of 4103 features representing 2 classes, i.e., normal and tumor.

(8) $$FV_{GLCM\;\left(2\times 7\right)}=\left(GLCM_{2\times 1},\;GLCM_{2\times 2},\ldots ,GLCM_{2\times 7}\right)$$

(9) $$FV_{VGG16\left(2\times 4096\right)}={\left(VGG16_{2\times 1\;\;},VGG16_{2\times 2},\ldots \;VGG16_{2\times 4096}\right)}_{\;}$$

(10) $$EV_{2x4103}=\overset{2}{\underset{i=1}{\sum }}{\left(FV_{VGG16},\;FV_{GLCM\;}\right)}_{\;}$$

### 3.3. Classification

Finally, EV is classified via ML-based classifiers, i.e., KNN and SVM. SVM belongs to the family of generalized linear classifiers that avoid overfitting data by maximizing its performance. It is a supervised learning approach developed by Vladimir Wapnik in 1992 [46]. KNN, on the contrary, was developed by Thomas Cover, a supervised learning algorithm deployed in problems related to regression and classification [47]. The algorithm is usually helpful in scenarios with little or no prior knowledge regarding data distribution. The classifier computes the distance between an individual training sample with specified (k) training samples and votes for the most frequent label in those *k* samples [48]. Both classifiers are adopted in classification and regression tasks because of promising results.

## 4. Results

### 4.1. Dataset

**Figure 3:**
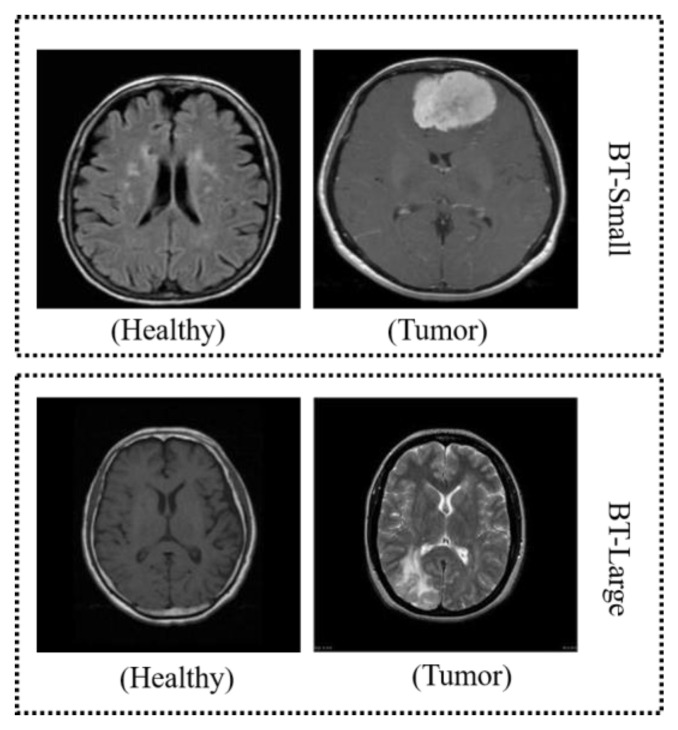
MRI samples from the employed datasets.

We conducted this study using two publicly available datasets of brain MR images for brain tumor classification. Both datasets are composed of normal and tumor MRIs. For ease, we named the first dataset BT-small [49] to conduct Study I and the second dataset BT-large to conduct Study II. The BT-small dataset comprises 253 MRI images, of which 155 are tumorous while the rest of the 98 images are normal. BT-large [50] is composed of 1500 normal images and 1500 tumor images. The sample images obtained from both datasets are displayed in Figure 3. We used 70% of the MRI samples to train the models and the remaining 30% of the samples for validation. Table 1 discusses the dataset in detail.

### 4.2. Evaluation Parameters

Since the aim of detection frameworks is to predict unexpected data, successfully assessing the model’s performance is crucial when developing automated systems. Thus, training and test sets evaluation illustrate the framework’s generalization abilities. The most common method for assessing a classification model is performed via a confusion matrix (CM), a simple cross-tabulation of the actual and classified data for each class. Accuracy, F1-score, recall, and precision are a few classification metrics based on the CM used to assess the model’s performance in this study. The F1-score is an effective tool for combining precision and recall in a single benchmark that contains features from both metrics. It is widely used in cases of data imbalance. The metrics used in this study, i.e., precision, recall, accuracy, and F1-score, are defined in Equations (11)–(14), respectively.

(11) Precision=TP/(TP+FP)

(12) Recall=TP/(TP+FN)

(13) Accuracy=(TP+TN)/(TP+TN+FP+FN)

(14) $$F1-\mathrm{Score}=\left(\mathrm{TP}\right)/(\mathrm{TP}+\frac{1}{2}\left(\mathrm{FP}+\mathrm{FN}\right))$$

TP, TN, FP, and FN stand for true positive, true negative, false positive, and false negative, respectively.

### 4.3. Results Obtained from the Proposed Framework

**Figure 4:**
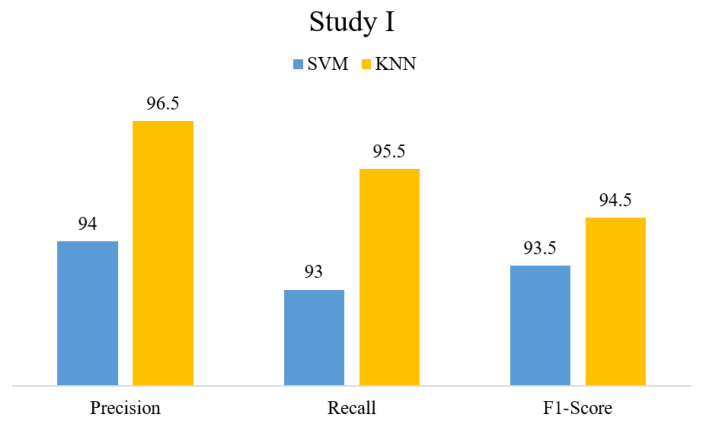
Study I Results in terms of PRE, REC, and F1-Score.

**Figure 5:**
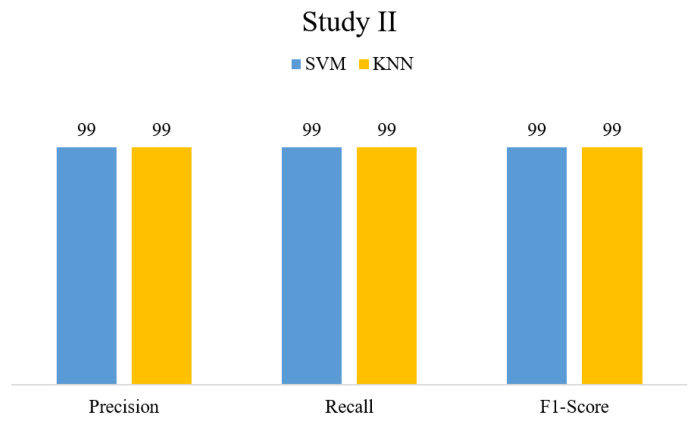
Proposed method results in terms of PRE, REC, and F1-Score.

In this section, we highlight the results obtained from the proposed method on two datasets, i.e., BT-small (Study I) and BT-large (Study II). Table 2 compares the proposed method performance for individual FVs and EVs. The results show that the classifiers performed better on the novel FV than on single-model FVs. In Study I, KNN classified the novel FV with 96% accuracy, whereas SVM obtained 93.3% accuracy. On the other hand, in Study II, SVM achieved a 99% classification accuracy, whereas KNN obtained 98.7% accuracy. Figure 4 and Figure 5 present precision, recall, and F1-score values obtained on EV for Study I and Study II, respectively.

**Figure 6:**
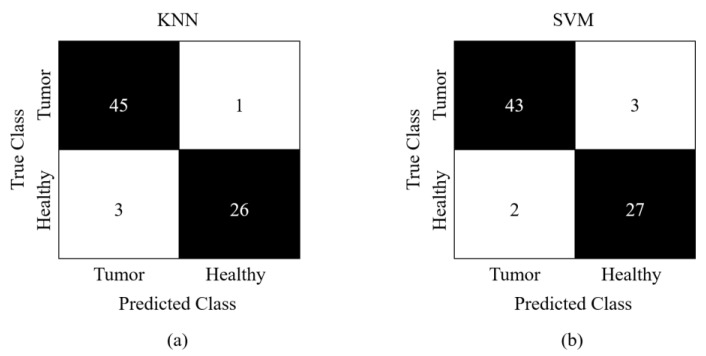
Confusion matrix obtained for Study I (**a**) KNN (**b**) SVM.

**Figure 7:**
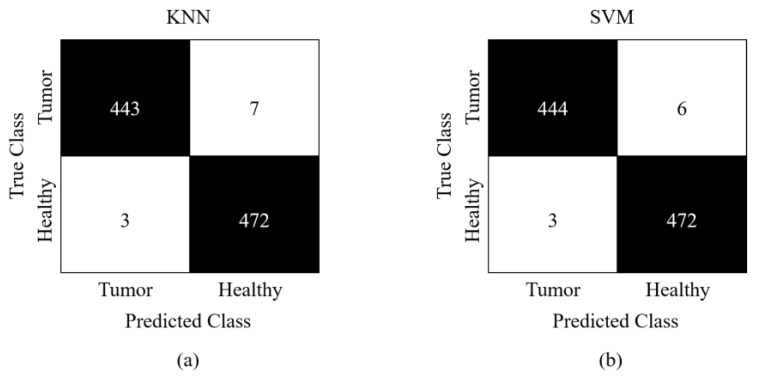
Confusion matrix obtained for Study II (**a**) KNN (**b**) SVM.

The classification performance of the proposed approach is shown in the CM in terms of actual and expected classes. The CM of the proposed FV obtained from Study I is presented in Figure 6. Here, (a) presents the CM obtained via KNN, while (b) shows the classification results for SVM. Study I obtains a classification accuracy of 96.0 using KNN and 93.3% using SVM, whereas, Figure 7 presents the CM of Study II. Part (a) denotes the CM obtained via KNN, and part (b) displays CM obtained via the SVM classifier. Study II obtains a classification accuracy of 98.7 using KNN and 99.0% using SVM. Study II achieved better performance due to a relatively larger database. These metrics demonstrate the reliability of our suggested strategy despite class imbalance because of a novel FV that contains discriminative and informative characteristics than an independent FV for the task of brain tumor classification.

**Figure 8:**
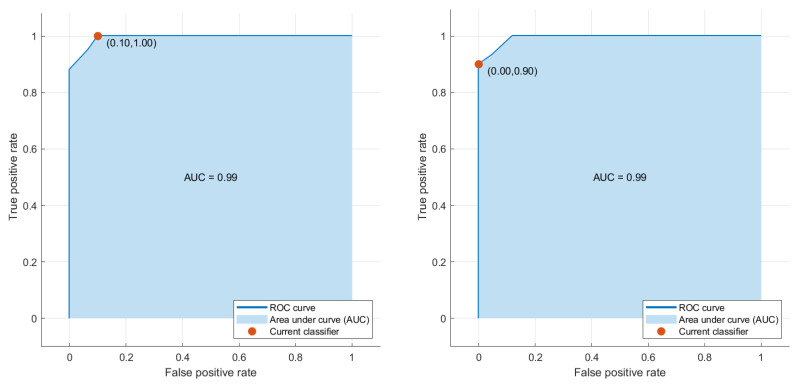
ROC Curve results obtained on BT-Small Dataset.

**Figure 9:**
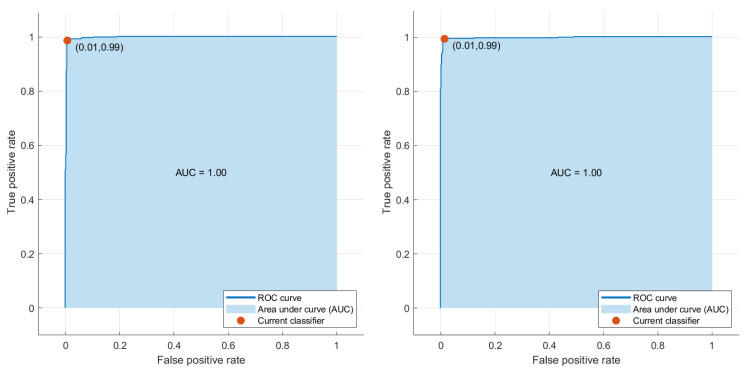
ROC Curve results obtained on BT-Large Dataset.

Figure 8 and Figure 9 show Reciever Operating Curves (ROC) obtained from BT-Small and BT-Large databases, respectively. The original purpose of the ROC analysis was to examine radar signal noise during World War II [51]. Over the past few decades, ROC curves have gained prominence as a tool for evaluating the effectiveness of medical diagnostic systems. The curve depicts the trade-off between sensitivity and specificity. The model is more accurate when ROC is positioned at the top left corner. It is worth mentioning fluctuations do not affect that accuracy index due to arbitrarily defined thresholds [52]. The area determines the discriminative capacity of a framework under the curve (AUC), demonstrating how effectively it operates in a specific scenario. A robust model will have almost one AUC [53]. It can be seen that the proposed framework succeeded in achieving efficient results as the AUC nears one on the BT-Small database, whereas AUC = 1 in the case of the BT-Large database due to the presence of more images.

### 4.4. Cross-Dataset Validation

We investigated the cross-dataset detection capability of our suggested technique. The primary goal of testing the model on cross-dataset scenarios is to assess the generalization potential of the suggested technique. We used BT-Small and BT-large datasets to train our approach, and we tested Study I’s performance using Study II’s dataset and vice versa. Detailed results of cross-dataset validation in terms of accuracy are presented in Table 3. Technique in Study I obtained 92% accuracy via SVM and 90.0% accuracy via KNN on the MRI images acquired from the BT-Large dataset. At the same time, Study II obtained 99.2% accuracy via KNN and 99.6% accuracy via SVM on MRI images from the BT-Small dataset. The classifiers in Study II performed better than those in Study I due to a larger MRI dataset. The results also show that our suggested methods can detect brain cancer in unseen MRI samples. This leads to the conclusion that the suggested strategy is reliable and effective for locating and classifying brain malignancies from MRI data.

### 4.5. Comparison with Existing Approaches

Recent medical image processing software developments have made it easier for medical professionals to identify ailments early on. These developments are helping them in some medical specialties, including disease diagnosis, as well as decision-making for clinical applications. Healthcare centers generate tons of medical records daily. Doctors and scientists looking for the best ways to utilize these ever-growing volumes of data effectively are helped by medical informatics research [54,55]. Early discovery and adequate treatment choices are critical for effectively treating brain tumor disorders. These treatment options are based on the type of tumor and its stage during detection time. The traditional identification methods use simple ML-oriented algorithms that only extract a few features [56]. This study introduces a unique feature ensemble-based method for precisely classifying brain cancers from MR images.

This paper presents a unique FV-based ensemble of hand-engineered and deep features for brain tumor classification. We investigated the usefulness of the proposed model by comparing our approach to existing brain tumor classification techniques in Table 4. Irsheidat et al. [57] proposed a method for detecting brain tumors using artificial CNNs. They trained a deep CNN on a dataset of MRI images to differentiate between normal and tumor images. The proposed method employed data augmentation techniques to increase the training dataset’s size and improve the model’s generalization. The authors reported an accuracy of 96.9% in detecting brain tumors using their proposed deep CNN model. However, the system attained lower accuracy than the proposed method.

On the contrary, Mask RCNN was employed by Masood et al. [21] to detect brain malignancies in MRI images. They used bounding boxes to pinpoint the tumor site in the first stage and then classified the tumors. Due to the need for tumor segmentation before the classification phase, this technique is computationally intensive. Kesav et al. [33] proposed an architecture for detecting and classifying brain tumors using a combination of region-based CNN and two-channel CNN. The proposed architecture used RCNN to identify regions of interest in MRIs and then fed these regions into a two-channel CNN for classification. The two-channel CNN comprises two separate CNN models, one for the segmentation of the tumor region and the other for the classification of the segmented tumor. However, the system is computationally expensive. Sharma et al. [56] used the concept of transfer learning for brain tumor detection. They employed VGG-16 architecture that attained 94.&% accuracy on the Kaggle database. Compared to previous methods, our suggested method is efficient, using a hybrid feature set consisting of deep features and GLCM features to identify and categorize brain tumors. Moreover, the proposed framework does not require segmentation before categorization. The ensemble of FVs obtained from deep and statistical methods results in a more discriminative feature representation than a single model FV. The proposed EV achieved the highest accuracy of 96% in Study I and 99% in Study II. From the results, it is evident that the proposed structure is robust and provides better classification results compared to existing studies.

## 5. Conclusions

This paper proposes a novel brain tumor detection and classification method using ensemble learning that integrates hand-crafted and deep features in a single FV. The hand-crafted are computed via GLCM, whereas the deep features are extracted from VGG-16. Both the independent FVs are then serially combined, resulting in a single FV (called EV) that is more discriminate and informative because of the concatenation of textural and deep features. EV is then supplied to well-known classification frameworks such as SVM and KNN. Our method is individually trained and validated on two benchmark databases and obtained maximum accuracy of 96% on the BT-Small dataset and 99% on the BT-Large database. We also validated the performance of our model via cross-dataset validation and compared the proposed technique with the existing systems. The results show the robustness of the proposed method and can be deployed in the real environment to detect brain tumors from MRI images accurately. In the future, we will gather medical images of other modalities and employ other CNN architectures to make the system more efficient. Furthermore, we are willing to test the proposed approach to perform the distinctive category-wise distribution of brain MRI images to determine the specific type of tumors as well.

## Figures and Tables

**Table 1:** Dataset Distribution.

Method	Dataset	Training Samples	Validation Samples
Study I	BT-small	177	76
Study II	BT-large	2100	900

**Table 2:** Proposed method results in terms of accuracy.

FV	Study I Accuracy %		Study II Accuracy %	
	SVM	KNN	SVM	KNN
**VGG16**	92.1	88.1	98.0	97.8
**GLCM**	72.0	84.0	96.1	96.0
**GLCM + VGG16**	93.3	96.0	99.0	98.7

**Table 3:** Cross Dataset Validation.

Classifiers/ Method	Accuracy %	
	Study I	Study II
SVM	92.0	99.6
KNN	90.0	99.2

**Table 4:** Comparison with existing techniques.

Reference	Technique	ACC(%)
Irsheidat et al. [32]	CNN	96.7
Kesav et al. [33]	Two Channel CNN	98
Tazin et al. [57]	Mobile Net V2	92
Sharma et al. [56]	VGG-19	94.7
Masood et al. [21]	Mask RCNN	98.3
**Proposed Study I**	**VGG16 + GLCM**	**96.0**
**Proposed Study II**		**99.0**

## Data Availability

Not applicable.
